# TRPA1 Modulating C_14_ Polyacetylenes from the Iranian Endemic Plant *Echinophora platyloba*

**DOI:** 10.3390/molecules23071750

**Published:** 2018-07-17

**Authors:** Giuseppina Chianese, Carmina Sirignano, Yalda Shokoohinia, Zeynab Mohammadi, Leili Bazvandi, Fataneh Jafari, Fereshteh Jalilian, Aniello Schiano Moriello, Luciano De Petrocellis, Orazio Taglialatela-Scafati, Daniela Rigano

**Affiliations:** 1 Department of Pharmacy, School of Medicine and Surgery, University of Naples Federico II, Via D. Montesano, 49, 80131 Napoli, Italy; g.chianese@unina.it (G.C.); carmina.sirignano@unina.it (C.S.); drigano@unina.it (D.R.); 2 Pharmaceutical Sciences Research Center, School of Pharmacy, Kermanshah University of Medical Sciences, Kermanshah 67571, Iran; fataneh.jafari@yahoo.com (F.J.); fe.jalilian@gmail.com (F.J.); 3 Student Research Committee, School of Pharmacy, Kermanshah University of Medical Sciences, Kermanshah 67571, Iran; zeynabmohamadi47@yahoo.com (Z.M.); leilibazvandi@yahoo.com (L.B.); 4 Epitech Group SpA, Saccolongo, 35030 Padova, Italy; aniello.schianomoriello@icb.cnr.it; 5 Endocannabinoid Research Group, Institute of Biomolecular Chemistry, Consiglio Nazionale delle Ricerche, Via Campi Flegrei 34, 80078 Pozzuoli, Italy; luciano.depetrocellis@icb.cnr.it

**Keywords:** *Echinophora platyloba*, polyacetylenes, echinophorins, TRPA1, inflammation

## Abstract

Phytochemical investigation of the apolar extract obtained from aerial parts of the Iranian endemic plant *Echinophora platyloba* DC (Apiaceae) resulted in the characterization of the polyacetylene fraction of this plant. This resulted to be composed of the known echinophorins A and B, embedding the very rare α-pyrone terminal, and of the new echinophorin D (**3**), including also three conjugated triple bonds. The chemical structures of these compounds were secured by detailed inspection of MS and 1D/2D NMR spectra. The isolated polyacteylenes were evaluated for their modulation of six thermo-TRP channels and they revealed a selective activity on TRPA1, an ion channel involved in the mediation of neuropathic and inflammatory pain. This is the first report on the activity of plant polyacetylenes on transient receptor potential (TRP) channels.

## 1. Introduction

*Echinophora* is a genus of the Apiaceae family (tribe Echinophoreae) widely distributed in the Mediterranean regions, from Spain to the Balkans, and the Middle East. As suggested by the Latin name (echino = spine, phora = leaf), these herbs are characterized by spiny leaves and yellow flowers and their preferred habitat is sandy coastal areas. Due to the pleasant aromatic taste, some *Echinophora* species have found use as seasoning in the preparation of food or as a flavoring agent for soups, meats, and dairy products [1]. Fresh or dried herbs are also employed in the traditional medicine, not only for the claimed antimicrobial and antifungal activities [2], but also for their wound healing properties, especially applied to treat gastric ulcers [3].

Among the about 20 *Echinophora* species described to date, only *E. cinerea* (Boiss.) Hedge et Lamond and *E. platyloba* DC are endemic to Iran. *E. platyloba* is a perennial aromatic plant, called “Khousharizeh” or “Tigh Touragh” in Persian, and commonly used as an edible vegetable [4]. The aerial parts and essential oils of the plant are also used by local people as anti-mold agent to preserve the quality of food and as folk remedy for several ailments, the main applications in traditional medicine being related to antispasmodic, diuretic, and antimicrobial activities [4,5,6].

Although a direct correlation between claimed biological activity and secondary metabolite content is still lacking, the *Echinophora* genus has been the object of intense phytochemical investigations that have disclosed the presence of saponins, flavonoids [7] and polyacetylenes [8].

Polyacetylenes constitute a large group of oxylipins that include at least two, usually conjugated triple carbon-carbon bonds. These compounds are produced thanks to the enzyme acetylenase, able to catalyze the conversion of a double bond into a triple bond via two subsequent hydrogen abstractions, following a mechanism that has been studied in detail [9]. Plants of the Apiaceae and Asteraceae families are undoubtedly the most prolific producers of polyacetylenic compounds [10], mainly concentrating them in the roots, although they have been frequently reported also from the aerial parts [10].

**Figure 1:**
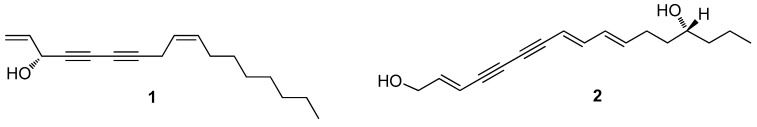
The chemical structures of two archetypal C_17_ polyacetylenes, falcarinol (**1**) and oenanthotoxin (**2**).

The most prominent class of polyacetylenes are undoubtedly C_17_ derivatives, exemplified by falcarinol (**1**) (Figure 1) [10], characterized by two triple bonds, two double bonds and a stereogenic hydroxymethine group. Several biological activities have been ascribed to these compounds, including antibacterial and antifungal activities, anticancer and anti-inflammatory properties at non-toxic concentrations for humans [11]. We have reported that another member of this family, oenanthotoxin (**2**), potently blocks GABAergic responses, thus providing an explanation for the symptoms of poisoning from water-dropwort (*Oenanthe crocata*) and related plants [12].

Conversely, the class of C_14_ polyacetylenes is much less widespread and, among them, even less common are natural polyynes containing an α-pyrone moiety. To our knowledge, this class of molecules counts only four members, having been isolated exclusively from *Mediasia macrophylla* [13] (in the glycosylated form) and, recently, from *Echinophora cinerea* [14].

**Figure 2:**
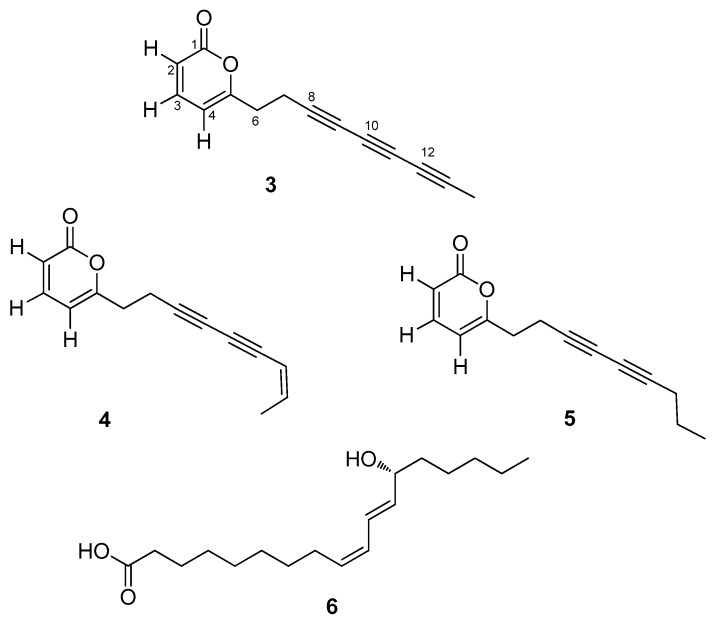
The chemical structures of **3**–**6**, isolated from *E. platyloba.*

In the frame of our research project aimed at discovering the medicinal potential of Iranian endemic plants [15,16], we have investigated the aerial parts of *E. platyloba* DC, for which only fragmentary phytochemical characterization is available in the literature. These studies were almost exclusively focused on the volatile fraction and the sterol composition [17] and reported a promising antifungal activity for the essential oils of the plant [4]. Herein, we report the isolation of polyacetylene derivatives (**3**–**5**) from *E. platyloba*, which includes the new secondary metabolite echinophorin D (**3**), along with the known **4** and **5** (Figure 2). These compounds were evaluated for their interaction with the transient receptor potential (TRP) channels of ankyrin type-1 (TRPA1), cation channels widely expressed in the oral and nasal cavity, which play an important role in the perception of nociceptive and inflammatory pain [18].

## 2. Results and Discussion

Aerial parts of *E. platyloba* were collected in the area of Marivan, Iran, and were dried and extracted sequentially with *n*-hexane, dichloromethane and acetone. Repeated purifications of the dichlorometane fraction by column chromatography and HPLC led to the isolation of pure compounds **3**–**5**. The structures of the known echinophorins B (**4**) and A (**5**) were secured by comparison of their spectroscopic data with those available in the literature [14]. The acetone fraction was found to contain the non-polyacetylene oxylipin coriolic acid (**6**) [19]: To our knowledge, this is the first report of coriolic acid from a plant of the Apiaceae family.

**Figure 3:**
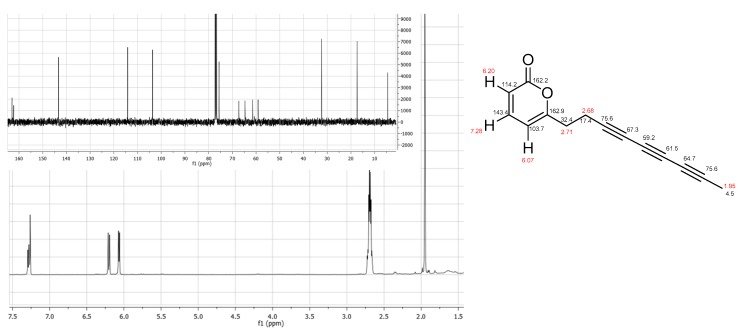
^1^H NMR (**bottom**) and ^13^C NMR (**top**) spectra of Echinophorin D (structure with NMR assignments on the right).

Echinophorin D (**3**) was isolated as a brown oil with molecular formula C_14_H_10_O_2_, as deduced on the basis of HR-ESIMS data (found *m*/*z* 233.0583, C_14_H_10_O_2_Na requires 233.0578). The ^1^H NMR spectrum of **3** (Table 1, Figure 3) was very simple showing two doublets at δ_H_ 6.20 (*J* = 9.2 Hz) and 6.07 (*J* = 6.8 Hz), and a double doublet at 7.28 (*J* = 9.2 and 6.8 Hz), two coupled methylenes between δ_H_ 2.65 and 2.75 and a methyl singlet resonating at δ_H_ 1.95. All these proton resonances were associated to those of the directly attached carbon atoms by means of the 2D NMR HSQC spectrum, thus disclosing the marked high-field resonance of the methyl carbon (δ_C_ 4.5).

In agreement with the molecular formula, the ^13^C NMR spectrum of **3** showed the resonances of eight non-protonated carbons, two of which were coincident at δ_C_ 75.6. The α-pyrone ring was defined on the basis of the HMBC correlations H-2/C-1, H-3/C-5 and H-7/C-5 and on the basis of the comparison of H/C resonances with NMR data of echinophorins A and B [14]. The assignment of the carbon resonances of the three conjugated triple bonds was achieved through inspection of the HMBC spectrum. In particular, the ^2^*J*_C-H_ and ^3^*J*_C-H_ correlations of Me-14 (δ_H_ 1.95) to C-12 (δ_C_ 64.7) and C-13 (δ_C_ 75.6) and of H_2_-7 (δ_H_ 2.68) with C-8 (δ_C_ 75.6) and C-9 (δ_C_ 67.3) led to the assignment of resonances for two of the three triple bonds. Since weak peaks for longer correlations (namely ^4^*J*_C-H_) can be observed for proton-deficient molecules embedding extensively conjugated systems, we could complete the assignment of the carbon resonances of the acetylenic system taking advantage of the HMBC cross-peaks from Me-14 to C-11 (δ_C_ 61.5) and H_2_-7 to C-10 (δ_C_ 59.2). Thus, the structure of **3** was unambiguously determined as the first polyacetylenic compound to include an α-pyrone system and three conjugated triple bonds.

The transient receptor potential (TRP) proteins are non-selective cation channels (permeable to both monovalent and divalent cations) ubiquitous in the human organism, where they act as regulators for many cell functions, including in the mediation of pain, taste, hot or cold sensations [20]. About 30 mammalian TRP channels have been identified and grouped into six main subfamilies: TRPC (canonical), TRPA (ankyrin), TRPV (vanilloid), TRPM (melastatin), TRPP (polycystin), TRPML (mucolipin). The transient receptor potential ankyrin 1 (TRPA1) channel, along with TRPV1 and TRPM8, is one of the most intensely investigated TRP channels since it plays a major role in noxious cold perception, and is involved in neuropathic and inflammatory pain [21], qualifying as a target for the discovery of novel analgesic and anti-inflammatory agents.

Natural products have a long history as TRP ligands [22] and many TRP channels are activated/antagonized by secondary metabolites: For example, allyl isothiocyanate and cinnamaldehyde [23] are activators of TRPA1, menthol and eucalyptol activate the cold receptor TRPM8 [24], etc. However, since new TRP modulators are needed as viable candidates for drug discovery, natural product ligands are still intensely investigated as leads for TRP channels deorphanization.

We have recently reported the identification of some TRPA1 ligands from natural sources, e.g., curcumin [25] and leucettamols [26], and two-headed sphingoid-like compounds. In the frame of this research project, we have decided to evaluate the three polyacetylenic compounds from *E. platyloba* **3**–**5** against six thermo-TRP channels (TRPA1, TRPV1, TRPV2, TRPV3, TRPV4, TRPM8) of great biomedical relevance. Coriolic acid (**6**) was not evaluated due to its small quantity.

While the overall activity on the remaining TRPs was negligible, we found that the three compounds were able to modulate TRPA1 (detailed results are reported in Table 2). Using a fluorimetric test, we observed that rat TRPA1-HEK293 cells exhibited an increase in intracellular [Ca^2+^]_i_ upon application of **3**–**5**. All compounds showed a discrete potency in the range 20–30 μM with echinophorin D, bearing three conjugated triple bonds, being the less potent compound of the series, and the two analogues showing two triple bonds exhibited an activity of almost the same magnitude. The activity of the compounds was normalised to the maximum intracellular Ca^2+^ elevation generated by application of allylisothiocyanate (AITC) 100 μM. Echinophorins A (**5**) and B (**4**) were able to reach about 80% of the maximal response of AITC, while the less active echinophorin D (**3**) reached only about 52%.

## 3. Discussion

Although the results obtained in this investigation are preliminary, since they derive from a very limited panel of natural products, they appear nevertheless interesting for a couple of reasons. Firstly, to the best of our knowledge, this is the first report describing the modulation of TRP channels by polycetylenic compounds. Secondly, what is also remarkable is the selectivity of echinophorins toward TRPA1, an endpoint of relevance for treatment of inflammation and pain. Several polyacetylenic compounds have shown anti-inflammatory effects, mainly related to inhibition of NF-κB or to the modulation of prostaglandin catabolism [27]. Our results suggest that modulation of TRPA1 could be another anti-inflammatory mechanism of polyacetylenes worthy of being explored.

## 4. Materials and Methods

### 4.1. General Experimental Procedures

^1^H (500 MHz) and ^13^C (125 MHz) NMR spectra were measured on a Varian INOVA spectrometer. Chemical shifts were referenced to the residual solvent signal (CDCl_3_: δ_H_ 7.26, δ_C_ 77.0). Homonuclear ^1^H connectivities were determined by the COSY experiment; one-bond heteronuclear ^1^H-^13^C connectivities by the HSQC experiment; two- and three-bond ^1^H-^13^C connectivities by gradient-HMBC experiments optimized for a ^2,3^*J* of 8 Hz. Low- and high-resolution ESI-MS spectra were performed on a LTQ OrbitrapXL (Thermo Scientific, Waltham, MA, USA) mass spectrometer. Separations were monitored by TLC on Merck (Kenilworth, NJ, USA) 60 F254 (0.25 mm) plates and were visualized by UV inspection and/or staining with 5% H_2_SO_4_ in ethanol and heating. HPLC were achieved on a Knauer (Berlin, Germany) apparatus equipped with a refractive index detector. LUNA normal phase SI60 (Phenomenex, Torrance, CA, USA) analytical (250 × 4 mm) and semipreparative (250 × 8 mm) columns were used, with 0.7 mL/min or 2.5 mL/min as flow rate.

### 4.2. Plant Material

The aerial parts of *E. platyloba* were collected in May 2010 on Mount Abidar, Marivan, Iran. The species was identified by Dr. Sayed Mohammad Masoumi, Razi University, and compared to the herbarium sample No. RUH585, Hamedan Herbarium, Iran.

### 4.3. Extraction and Isolation

Aerial parts of *E. platyloba* (dry weight, 456 g) were extracted subsequently with *n*-hexane, dichloromethane and acetone using Soxhlet apparatus, 4 h for each. The hexane phase (4.85 g) was composed mainly of fats and fatty acids and was no further investigated. The dichloromethane extract (4.98 g) was dissolved in MeOH (60 mL) and stored at −20 °C for 2 days. Afterwards, it was filtered chilled and the filtrate was fractionated by gravity column on RP18 stationary phase, using a solvent gradient from MeOH:H_2_O 1:1 to MeOH to obtain six fractions. The fraction eluted with MeOH/H_2_O 7:3 (62.0 mg) contained polyacetylenes, by ^13^C NMR of the crude fraction, and was therefore separated by HPLC (*n*-hexane/EtOAc 1:1, flow rate 0.7 mL/min) to afford pure echinophorin B (**4**, 5.1 mg), echinophorin A (**5**, 1.3 mg) and echinophorin D (**3**, 24.3 mg). The acetone extract (4.75 g) was fractionated by gravity column on silica gel using a gradient of heptane/EtOAc of increasing polarity (from heptane/EtOAc 9:1 to 1:1) to get eight fractions. The fraction eluted with heptane/EtOAc 7:3 (150 mg) (was purified by HPLC (*n*-hexane/EtOAc 7:3, flow rate 0.8 mL/min) to yield coriolic acid (**6**, 0.8 mg).

### 4.4. Echinophorin D (**3**)

Brown oil. ^1^H NMR (500 MHz): Table 1; ^13^C NMR (125 MHz): Table 1. ESI-MS (positive ions): *m/z* 233 [M + Na]^+^; HRESI-MS: found *m*/*z* 233.0583, C_14_H_10_O_2_Na requires 233.0578.

### 4.5. Thermo-TRPs (TRPV1, TRPV2, TRPV3, TRPV4, TRPM8, TRPA1) Receptor Assays

HEK-293 cells stably over-expressing recombinant rat TRPA1, TRPM8, TRPV2-4, TRPM8 or human TRPV1 were selected by Geneticin 600 μg mL^−1^, grown on 100-mm diameter Petri dishes as monolayers in minimum essential medium supplemented with non-essential amino acids, 10% fetal bovine serum, and 2 mM glutamine, and maintained under 5% CO_2_ at 37 °C. Stable expression of each channel was checked by quantitative real time-PCR. The effect of the substances on intracellular Ca^2+^ concentration [Ca^2+^]_i_ was determined using Fluo-4, a selective intracellular fluorescent probe for Ca^2+^. Toward this aim, on the day of the experiment, cells over-expressing the TRP channels were loaded for 1 h in the dark at room temperature with the methyl ester Fluo4-AM (4 μM in DMSO containing 0.02% Pluronic F-127, Invitrogen, Carlsbad, CA, USA) in minimum essential medium without fetal bovine serum. After the loading, cells were washed twice in Tyrode’s buffer (145 mM NaCl, 2.5 mM KCl, 1.5 mM CaCl_2_, 1.2 mM MgCl_2_, 10 mM d-glucose, and 10 mM HEPES, pH 7.4), re-suspended in Tyrode’s buffer, and transferred (about 100,000 cells) to the quartz cuvette of the spectrofluorimeter (Perkin-Elmer LS50B; PerkinElmer Life and Analytical Sciences, Waltham, MA, USA) under continuous stirring. [Ca^2+^]_i_ was determined before and after the addition of various concentrations of test compounds by measuring cell fluorescence at 25 °C (λ_EX_ = 488 nm, λ_EM_ = 516 nm). Curve fitting (sigmoidal dose-response variable slope) and parameter estimation were performed with GraphPad Prism^®^ (GraphPad Software Inc., San Diego, CA, USA). Potency was expressed as the concentration of test substances exerting a half-maximal agonist effect (i.e., half-maximal increases in [Ca^2+^]_i_ (EC50), calculated by using GraphPad^®^. The efficacy of the agonists was first determined by normalizing their effect to the maximum Ca^2+^ influx effect on [Ca^2+^]_i_ observed with application of 4 μM ionomycin (Sigma, St. Louis, MO, USA). The increases in fluorescence in wild-type HEK293 cells (i.e., not transfected with any construct) were used as baseline and subtracted from the values obtained from transfected cells. The effects of TRPA1 agonists are expressed as a percentage of the effect obtained with 100 μM allyl isothiocyanate (AITC). Antagonist/desensitizing behavior was evaluated for TRPA1 by adding the test compounds in the quartz cuvette 5 min before stimulation of cells with the agonist AITC (100 μM). Data are expressed as the concentration exerting a half maximal inhibition of agonist-induced [Ca^2+^]_i_ elevation (IC_50_), which was calculated again using GraphPad Prism^®^ software. The effect on [Ca^2+^]_i_ exerted by agonist alone was taken as 100%. Dose-response curves were fitted by a sigmoidal regression with variable slope. All determinations were at least performed in triplicate. Statistical analysis of the data was performed by analysis of variance at each point using ANOVA followed by Bonferroni’s test.

## Figures and Tables

**Table 1:** ^1^H and ^13^C NMR data of Echinophorin D (**3**) ^a^.

Pos.	δ_H_, Mult., *J* in Hz	δ_H_, Mult.
1		162.2, C
2	6.20, d, 9.2	114.2, CH
3	7.28, dd, 9.2, 6.8	143.4, CH
4	6.07, d, 6.8	103.7, CH
5		162.9, C
6	2.71, m	32.4, CH_2_
7	2.68, m	17.4, CH_2_
8		75.6, C
9		67.3, C
10		59.2, C
11		61.5, C
12		64.7, C
13		75.6, C
14	1.95, s	4.5, CH_3_

^a^ Spectra registered in CDCl_3_.

**Table 2:** Activity of compounds **3**–**5** on calcium influx in HEK293 cells transfected with rTRPA1.

Compounds	Efficacy ^a^	Potency EC_50_ μM	IC_50_ inh TRPA1 μM (AITC 100 μM)
Echinophorin D (**3**)	51.7 ± 1.3	30.9 ± 2.8	87.0 ± 1.5
Echinophorin B (**4**)	82.0 ± 2.8	25.0 ± 3.0	37.2 ± 0.8
Echinophorin A (**5**)	81.0 ± 3.3	20.3 ± 3.2	45.7 ± 3.5

^a^ % AITC (Allylisothiocyanate) at 100 μM, used as a control.
